# Supplementary material for: Prevalence and factors associated with anaemia among pregnant women attending reproductive and child health clinics in Mbeya region, Tanzania
Source: PLOS Glob Public Health. 2022 Oct 5;2(10):e0000280. doi: 10.1371/journal.pgph.0000280 (PMC10021601; doi:10.1371/journal.pgph.0000280)
Supplement: Attachment — Submitted filename: A rebuttal letter.doc [file pgph.0000280.s001.doc]

A rebuttal letter that responds to each point raised by the editor and reviewer(s)

**A: Response to the editor**

**Editor General Comment**: *Thank you for submitting your manuscript to PLOS Global Public Health. After careful consideration, we feel that it has merit but does not fully meet PLOS Global Public Health’s publication criteria as it currently stands. Therefore, we invite you to submit a revised version of the manuscript that addresses the points raised during the review process*

**Response: Thank you for coordinating the review process. We appreciate the time and effort that you and the reviewers have dedicated on providing invaluable comments and suggestions on this manuscript. We have revised the manuscript and addressed reviewer’s comments.**

**B: Additional Editor Comments 1:** *Please include recent national and international data on prevalence and risk factors for anemia if they are available. The study rationale should be more clearly stated in the introduction.*

**Response 1: We agree with this comment and added recent published national and international data on prevalence and risk factors for anemia. Reference number 13, 22, 24, 29, 33,38, 39, 40 and 41.Further, the study rationale has been clearly stated in paragraph 4 of the background section, line number 38- 41.**

**Additional Editor Comments 2:** *Consider adding a section detailing how the questionnaire was designed or at minimum provide an overview of the study variables including details about the diet or food intake ones especially (but the other major ones as well).*

**Response 2: Thank you very much for pointing this out. We agree with this comment and incorporate your suggestions in a methods section, line number 91-125.**

**Additional Editor Comments 3:** *If the authors have the sample broken down by health care facility please include this in the text or table. There are some typographic errors that the reviewers have detailed require correction (in the manuscript abstract, main text and tables). Please check to ensure the variable categories and spellings are appropriate.*

**Response 3: Thank you very much for pointing this out. We agree with this comment and incorporate your suggestions in a methods section, line number 57-62. Also, we have addressed all typographic errors as suggested.**

**Additional Editor Comments 4*:*** *Are the wealth quintiles in the correct order, and if so, how do the authors explain the results of greater anemia with higher wealth? Include the results of the bi-variate assessments in the table (unadjusted ORs). Further discussion of underlying factors that might be responsible for the study findings and the implications of the findings are required.*

**Response 4: Thank you very much for pointing this out. We agreed with comment and re-arrange the wealth quintiles in a correct order (see table 1, 2 and 3). The bi-variate assessments have been included in table 2 (unadjusted ORs). Further, we have included variables for smoking and drinking habits as suggested (see table 1 and 2).**

**C: Journal Requirements:**

**Comment 1**: *Please amend your detailed Financial Disclosure statement. This is published with the article, therefore should be completed in full sentences and contain the exact wording you wish to be published.
ii). State the initials, alongside each funding source, of each author to receive each grant.
iii). State what role the funders took in the study. If the funders had no role in your study, please state: “The funders had no role in study design, data collection and analysis, decision to publish, or preparation of the manuscript.”*

**Response 1: We agree with this comment and rephrase the financial disclosure statement as follows “This research received grants from the United Nations Children’s Fund (UNICEF) –Tanzania.**

**The funders had no role in study design, data collection and analysis, decision to publish, or preparation of the manuscript”.**

**Comment 2**: *Please include the Funding Information in the system. This should have the same information of Financial Disclosure statement*.

**Response 2: We agree with this comment and rephrase the Funding Information in the system as the same as information of Financial Disclosure statement.**

**Comment 3:** *Please amend your Data Availability Statement and indicate where the data may be found*

**Response 3: We agree with this comment and rephrase the statement as ‘all datasets underlying this study are freely available at the public repository ‘osf.io/7ysb9. 2021’.**

**Comment 4**: *Please review your reference list to ensure that it is complete and correct. If you have cited papers that have been retracted, please include the rationale for doing so in the manuscript text, or remove these references and replace them with relevant current references. Any changes to the reference list should be mentioned in the rebuttal letter that accompanies your revised manuscript. If you need to cite a retracted article, indicate the article’s retracted status in the References list and also include a citation and full reference for the retraction notice.*

**Response 4: Thank you very much for pointing this out. We agree with this comment and revise our references list (see line number 300-410).** *.*

1. **Comments to the Author**  *Does this manuscript meet PLOS Global Public Health’s* [*publication criteria*](https://journals.plos.org/globalpublichealth/s/criteria-for-publication)? *Is the manuscript technically sound, and do the data support the conclusions? The manuscript must describe methodologically and ethically rigorous research with conclusions that are appropriately drawn based on the data presented.*

*Reviewer #1: Yes*

*Reviewer #2: Yes*

**Response: Thank you very much for your consideration.**

2. **Comments to the Author**

*Has the statistical analysis been performed appropriately and rigorously?*

*Reviewer #1: Yes*

*Reviewer #2: Yes*

**Response: Thank you very much for your consideration.**

3. **Comments to the Author**

*Have the authors made all data underlying the findings in their manuscript fully available (please refer to the Data Availability Statement at the start of the manuscript PDF file)?
The PLOS Data policy requires authors to make all data underlying the findings described in their manuscript fully available without restriction, with rare exception. The data should be provided as part of the manuscript or its supporting information, or deposited to a public repository. For example, in addition to summary statistics, the data points behind means, medians and variance measures should be available. If there are restrictions on publicly sharing data—e.g. participant privacy or use of data from a third party—those must be specified.*

*Reviewer #1: No*

*Reviewer #2: Yes*

**Response: Thank you very much for your consideration.We agrees with these comments and rephrases the statement of data availability. ‘All datasets underlying this study are freely available at the public repository ‘osf.io/7ysb9. 2021’**

**4. Comments to the Author**

*Is the manuscript presented in an intelligible fashion and written in standard English?
PLOS Global Public Health does not copyedit accepted manuscripts, so the language in submitted articles must be clear, correct, and unambiguous. Any typographical or grammatical errors should be corrected at revision, so please note any specific errors here.*

*Reviewer #1: Yes*

*Reviewer #2: Yes*

**Response: Thank you very much for your consideration**

**D. Review Comments to the Author**

**Reviewer #1:** *Introduction*
*1. Introduction need to improve with recent data both nationally and internationally.
2. Authors needs to highlight the rational of the study clearly.*

**Response: Thank you very much for pointing this out. Introduction section has been improved with recent data both nationally and internationally (see line number 12-49). Further, the rationale has also been improved in paragraph 4 of the background section (line number 33-46).**

**Reviewer #1:** *Methodology
1. Data collection procedure needs to explain clearly.
2. Authors can add a section named Questionnaire design to explain the questionnaire part.
3. in data analysis section, clear outcome and predictor variable.*

**Response: Thank you very much and we agreed with these arguments. The data collection procedure has been improved and including the additional of subsection describes the questionnaire part (see line number 94-132). Further, the data analysis subsection has been improved and clearly indicate the outcome and predictor variables (line number 135-150).**

**Reviewer #1:** *Discussion
1.The discussion generally situates the present study within the broader literature in the study field, which is fine. What it falls short is that it does not really interpret the findings as to the possible underlying factors responsible for the study findings, the inferences from and the implications of the findings. To me, the discussion in its current form mainly compares, and rarely interprets, infers and implies.
2. The discussion of the study findings should be strengthened. Authors should do well to assign reasons for their findings. Authors should extensively discuss the implications of the study in terms of policy, practice and future research.*

**Response: Thank you very much for pointing this out and, we have revised the discussion as recommended (see line number 214-271).**

**Reviewer #2**: *Abdallah et al. have presented an interesting study on the prevalence and factors associated with anemia among pregnant women attending reproductive and child health clinics in Mbeya region, Tanzania. The study relies on the data from hospital-based surveys and reports that pregnant women with second and middle wealth quintiles were two times more likely to be anemic. The authors also have a well-written method section, especially the sampling part of data and process involved in the collection, and this study addresses some key questions. However, it will be great if the authors can have more clarity for the following points:
- I did not find the sample for every RCH facility, which means how many samples were collected from each RCH. The authors give the collected sample distribution by RCH in the appendix. That will be helpful to understand the cluster of anemic women because, in the introduction part, it mentions that geography also plays a role in anemia that means unavailability of health facilities or other reasons. Here I did not understand these facilities are public or private entities give some explanation.*

**Response: We agreed with this comments and we have added the statement of sample for every RCH facility in the methods section (see lines number 57-62). The variable of geographical location of sample collected per councils (RCHs) has been added (see table 2, 3 and 4).**

**Reviewer #2**: *What is food information had been collected is missing in the method section. As in the introduction section, it is mentioned that diet has an essential role in anemia, so the diet group must be in the study.
Further, what criteria of prime diet quality score are not come out.
Is PDQS similar to dietary diversity?
Why have authors categorized PQDS in only two groups?*

**Response: W agreed with comment. The information on food information has been improved and clearly highlights food components collected (see line number 94-114).**

**PDQS is the new index and is not exactly similar to dietary diversity (please refers the description of PDQS line number 94-114). PDQS is the prime diet quality score with 20 food groups: 13 healthy foods and 7 unhealthy foods. In our study we assess each of the 20 components of PDQS to investigate whether specific dietary components might explain an association with anaemia among pregnant women. PDQS components are measured in a likert scale and since our results were skewed, we decide to categorize into two groups at the medium.**

**Reviewer #2:** *Control variables were missing in the methods section includes it by which the reader can quickly come out the variable descriptions.
The information of outcome variables was also missing***.**

**Response: W agreed with comment and we have re-write the method section to clearly indicate the outcome and predictor variables.**

**Reviewer #2:** *In the result section (line no 6), the sentence “Out of the 240 respondents to the questionnaire,” is it 420 or 240” seems confusing*

**Response: Thank you very much for pointing this out. We have made a correction in that sentence “out of the 420 respondents……”**

**Reviewer #2:** *In table 1 and 2 need some modification in the categories of the following variables
(a) The age group should be in four categories (15-19, 20-24, 25-29, and ≥30 years).
(b) Marital status should be in three categories (Married, Cohabit, Single/Divorced)
(c) Anemia status (no, 10.0- 10.9 g/dl =mild, <7.0 – 9.9 g/dl= moderate) define in the variable’s description mild and moderate only. If it is binary, then keep in yes and no only.* **Response: We agreed with this comments and made modification in the categorization of age group, marital status and anaemia status as suggested (see table 1, 2, 3 and 4).Reviewer #2:** *In the result section “Prevalence of anemia in the study area,”; I have not found the table regarding the prevalence of anemia. Is it any supplementary tables? I also did not find the supplementary table. So, make it clear.
Further, the bivariate model is missing from the analysis, which is essential to understand the association between outcome and independent variables; keep it in table 1.* **Response: Thank you very much for pointing this out. We have created a new table specific for the distribution of anaemia (table 1). Also we have re-write the first paragraph of result section to make it clear. Furthermore, as suggested, we have added the bivariate model in table 3.**

**Reviewer #2:** *During interpretation of the result no need to give the p-value; CI is sufficient.
The multivariate analysis includes all control variables instead of only selected variables.*

**Response: We agreed with comments. For controlling of cofounders in the multivariate analysis, we only included variables that were significant at univariate analysis (table 3).**

**Reviewer #2:** *In the discussion section, there is no need to repeat all the findings; only explain in terms of higher or lower association. Add if any government policy is going on to improve the nutritional status of women***.**

**Response: Thank you very much and we agreed with comments. We have re-write the discussion section and try to remove the repetition of the findings. Further, we have added government policy that aims on improving the nutritional status of women.**

**Reviewer #2:** *Anemia is modifiable to explain the finding make it cautious. Further sample study only based on those women who were taking the health facility means they have some previous knowledge about health, hence write these are the terms in the limitation.
Please check the abstract result; the author has mentioned “less associated,” I think it will be higher or more likely. Similarly, check the spelling of variables in table 1***.**

**Response: Thank you very much for pointing this out and we agreed with comments and makes appropriate corrections**
