# Supplementary material for: Prevalence and factors associated with anaemia among pregnant women attending reproductive and child health clinics in Mbeya region, Tanzania
Source: PLOS Glob Public Health. 2022 Oct 5;2(10):e0000280. doi: 10.1371/journal.pgph.0000280 (PMC10021601; doi:10.1371/journal.pgph.0000280)
Supplement: Attachment — Submitted filename: A rebuttal letter02_Final.docx [file pgph.0000280.s002.docx]

Dear Colleen M. Davison,

Once again, thank you very much for giving us the opportunity to submit a second revised draft of our manuscript titled “Prevalence and factors associated with anaemia among pregnant women attending reproductive and child health clinics in Mbeya region, Tanzania” to *PLOS Global Public Health*.

We appreciate the time and effort that you have dedicated to providing your valuable feedback on our manuscript.

Here is a point-by-point response to the editor and reviewers’ comments and concerns.

**Comment 1:** *Please use the same spelling of “anaemia” throughout the study.*

**Response**: Thank you very much for pointing this out and incorporated your suggestion throughout the manuscript.

**Comment 2:** *In the revised manuscript line number 141, keep OR in brackets*

**Response:** We agreed with this comment and bracket the OR as suggested.

**Comment 3:** *Inline number 143, the author has used the “near” word. I think it would be “equal”.*

**Response:** We agreed with this comment and inserted ‘*equal*’ in line number 143

**Comment 4:** *No need to show the odds values in the discussion section*

**Response:** We agreed with this comment and remove the odd values in the discussion section

**Comment 5:** *Please do not include any statistics in the conclusion section*.

**Response**: Thank you very much for pointing this out and we have removed statistics in the conclusion section.

**Comment 6**: *In Table 2, the malaria variables sample is only 411, but I have read it was 420. So, please check it for all variables.*

**Response:** We agreed with this comment and incorporated your suggestion for all variables.

**Comment 7**: *In Table 2, the percentage values are exceeding 100 percent, so please check it and correct it.*

**Response:** We agreed with this comment and review all the percentage value in table 2.

**Comment 8**: *In Table 3, the not employed column total percentage is only 80% make sure it will 100%.*

**Response**: Thank you very much for pointing this out and we have corrected the percentage in the table 3

**Comment 9**: *In table 3, keep only “anaemic column”, no need for “not anaemic column”*

**Response:** We agreed with this comment and incorporated your suggestion in table 3.

Sincerely,

Ray Mrisho Masumo, PhD
