# Supplementary material for: Prevalence and factors associated with anaemia among pregnant women attending reproductive and child health clinics in Mbeya region, Tanzania
Source: PLOS Glob Public Health. 2022 Oct 5;2(10):e0000280. doi: 10.1371/journal.pgph.0000280 (PMC10021601; doi:10.1371/journal.pgph.0000280)
Supplement: Attachment — Submitted filename: A rebuttal letter2_final_Fatuma_04_08_2022.docx [file pgph.0000280.s003.docx]

Dear Dickson Abanimi Amugsi, PhD

Once again, thank you very much for giving us the opportunity to submit a revised draft of our manuscript titled “Prevalence and factors associated with anaemia among pregnant women attending reproductive and child health clinics in Mbeya region, Tanzania”
We appreciate the time and effort that you and reviewers have dedicated to providing valuable feedback on our manuscript.

Here is a point-by-point response to the editor and reviewers’ comments and concerns.

**General comments**

**Comment 1:** *It is OK to write aOR (adjusted Odds Ratio) after spelling it out at first use. Effect these changes throughout the manuscript.*

**Response**: We highly appreciate comment and, we have carefully made changes throughout the manuscript.

**Comment 2:** *Why did you use a power of 95% instead of 80% (a commonly used parameter).*

**Response:** Thank you very much for this comment; we have rephrased the paragraph for clarity (line number 72-77).

**Comment 3:** *Is the 1.5 the same as design effect? Clarify*

**Response:** Thank you very much for this comment; yes 1.5 is a design effect and so, we have rephrased the paragraph to enhance clarity (line number 74-75).

**Comment 4:** *Is the 420 stated in the preceding line inclusive of non-response rate of 10% in the second line. This appears a bit confusing*

**Response:** Thank you very much and, we highly appreciate the insightful and helpful comments. We have rephrased the paragraph for clarity (line number 72-77).

**Comment 5:** *Is the assumption of 0.10 as the difference between the two groups the same as intracluster correlation coefficient (ICC)?*

**Response**: Thank you very much for pointing out this. Indeed 0.10 was assumed as intracluster correlation coefficient (ICC).

**Comment 6**: *The study used two stage sampling strategy. This suggests that design effect must be accounted for in all the analysis. It appears this was not done. However, if it was accounted for, explain how it was done. And if it was not done, explain why.*

**Response:** Thank you very much for this comment. The design effect of 1.5 was assumed for accounting loss of information inherent in the clustered design.

As we know, design effect is a function of the average number of subjects sampled per cluster and of the intraclass correlation coefficient.

Design effect = 1 + (m-1) ICC

Where

- m is the average number of subjects sampled per cluster,
- ICC is the intraclass (or intracluster) correlation coefficient

**Comment 7***: The ethical statement needs a bit more details, especially regarding the consenting process e.g. Was the risks and benefits of the study explained to the participants? Were they informed of their rights to refuse to participate in the study or withdraw from it at any time, without consequences? What about the issues of confidentiality?*

**Response:** Thank you very much and we agreed with these arguments. We have rephrased the ethical statement (line number 162- 166).

**Comment 8***: Remove all p-values, ORs and CIs from the discussion section.*

**Response:** W agreed with this comment and, all the p-values, ORs and CIs were removed in the discussion section.

**Review Comments to the Author**

**Reviewer #3**

**Comment 1**: *Line 29-30; the sentence can be improved by rewording. Suggesting: During pregnancy, there is a marked increase in the minimum adult requirement by almost 2-3 folds for iron and 10-20 folds for folate*.
**Response:** We agreed with this comments and made corrections as suggested (line number 28-29).

**Comment 2:** *Line 88-89: Please mention the criteria for eligibility*

**Response:** Thank you very much and we agreed with comments. We have re-write the sentence and indicated the criteria for eligibility (line numbers 82-84).

**Comment 3**: *Line 232- Instead of despite of say “in spite of”*

**Response:** Thank you very much and we agreed with comment. We have inserted the word ‘in spite of’ instead of ‘despite’

**Comment 4**: *Line 233-238- No need to repeat results in discussion. Delete this: In our study (univariate level), anaemia was significantly associated with pregnant women who had attended a secondary education or higher, household wealth index (in poor and middle quintiles), received a supplementation of iron and folic acid, geographical location (Mbeya district council, Busekelo district council and Mbeya city council), as well as with the consumption of dark leafy green vegetables and, vegetable liquid oil*.

**Response**: Thank you very much for pointing this out and, the sentence was deleted.

**Comment 5:** *Line 238-239- The sentence ‘Generally, these findings are in consistent with the body of literatures that indicate that pregnancy increases the demand for iron, a demand that is often not met by food alone is confusing. Rephrase: Generally, these findings are consistent with the body of literature indicating that pregnancy increases the demand for iron, not met by food alone.*

**Response:** Thank you very much for pointing this out. We agreed with comment and re-phrased the sentence.

**Comment 6**: *Line 272-273- Rephrase: This is the first study to report the prevalence of anaemia and its predictors among pregnant women (less than 28 weeks of gestation) attending antenatal clinics in the Mbeya Region of Tanzania.*

**Response:** Thank you very much and we agreed with comments. We have rephrased the sentence.

**Comment 7**: Line 277 – Instead of casual say causal
**Response:** Thank you very much and we agreed with comments. We have inserted the word ‘causal’

**Comment 8:** *Line 288- Instead of risk factors say predictors*

**Response:** Thank you very much and we agreed with comments. We have inserted the word ‘predictors’ instead of risk factors.

**Reviewer #4
Comment 1:** *They need to re-analyze the parity and anemia relationship: Was there a relationship between anaemia and increasing parity? E.g. Nulliparity, para 1-2, para 3-4 then grand multipara*

**Response:** Thank you very much and we agreed with comments. We have re-analyzed the relationship between parity and anaemia as suggested (line number 179, table 2 and table 3)

**Comment 2**: *Were the different types of anaemia considered? E.g. Nutritional anaemia (iron deficiency, folate and B12 anaemia, sickle cell anaemia, etc)*

**Response**: Thank you very much and, we highly appreciate the insightful and helpful comments on our manuscript. This study only focused on nutritional anaemia though we acknowledged the importance of folate and B12 anaemia, sickle cell anaemia, etc.

**Comment 3**: *What was the criteria for taking the variables from univariate to multivariable analysis?*

**Response**: Thank you very much for pointing this out. The criteria was mentioned (line number 141-143) as: ‘Independent variables selected for the Multivariable logistic regression were included if they had a *P-value* equal to 0.05 in the univariate analysis’

**Comment 4**: *There are repetitions in the references: Eg. Reference 2 and 22, 5 and 6, 14 and 30, 25 and 29. Please organize your references according to the agreed criteria*.

**Response:** Thank you very much and we agreed with comments. We have removed all the repetitions and organized all references accordingly

**Reviewer #5**

**Comment 1**: *Please clarify here whether the model was conducted only with anemic women?*

**Response**: Thank you very much and, we highly appreciate the insightful and helpful comments on our manuscript. We agreed with comment and rephrase the sentence

**Comment 2**: *Please make sure that the reference number comes before the end of the phrase.*

**Response:** Thank you very much and we agreed with comments. We have ensured all reference number comes before the end of the phrase.

**Comment 3**: *sentence not clear. Could you please re-phrase it?*

**Response:** Thank you very much and we agreed with comments. We have reedited the sentence for clarity (line number 17).

**Comment 4**: *Please indicate whether the measurements were carried out in duplicate or not?*

**Response**: Thank you very much for pointing this out. We have added a sentence to indicate that measurements were carried in duplicate (line number 127).

**Comment 5**: *Please indicate in the methodology how did you deal with severely anemic women? Have you treated them or referred them?*

**Response:** Thank you very much for pointing this out. We have added this sentence ‘Severely anaemic pregnant women were referred immediately to the nearby health facility’ (line number 173-174).

**Comment 6:** *The same question regarding women who tested positive for malaria, were they treated?*

**Response:** Thank you very much for pointing this out. We have added this sentence ‘Pregnant women who tested positive for malaria were referred to the nearby health facility’ (line number 183-184).

**Comment 7**: *The results present the OR in all directions, that is it does not report clearly which risk factors are negatively associated (or positively associated)*

**Response:** Thank you very much and we agreed with comments. We have rephrased the paragraph (line number 193- 196).

**Comment 8:** *In this paragraph again, the factors that were positively or negatively associated are put all together which does not make sense*

**Response:** Thank you very much and we agreed with comments as also suggested by Reviewer #3 (comment 4).

**Comment 9**: *Disorders in hemoglobin can such as hemoglobinopathy can also be assessed with laboratory analyses.*

**Response:** Thank you very much and we agreed with comments. We have rephrased the sentence (line number 277).

Sincerely,

Ray Masumo, PhD
